# Growth Hormone Mitigates against Lethal Irradiation and Enhances Hematologic and Immune Recovery in Mice and Nonhuman Primates

**DOI:** 10.1371/journal.pone.0011056

**Published:** 2010-06-16

**Authors:** Benny J. Chen, Divino Deoliveira, Ivan Spasojevic, Gregory D. Sempowski, Chen Jiang, Kouros Owzar, Xiaojuan Wang, Diane Gesty-Palmer, J. Mark Cline, J. Daniel Bourland, Greg Dugan, Sarah K. Meadows, Pamela Daher, Garrett Muramoto, John P. Chute, Nelson J. Chao

**Affiliations:** 1 Department of Medicine, Duke University Medical Center, Durham, North Carolina, United States of America; 2 Department of Pathology, Duke University Medical Center, Durham, North Carolina, United States of America; 3 Department of Biostatistics and Bioinformatics, Duke University Medical Center, Durham, North Carolina, United States of America; 4 Department of Immunology, Duke University Medical Center, Durham, North Carolina, United States of America; 5 Human Vaccine Institute, Duke University Medical Center, Durham, North Carolina, United States of America; 6 RadCCORE Biostatistics Core, Duke University Medical Center, Durham, North Carolina, United States of America; 7 Section on Comparative Medicine, Wake Forest University School of Medicine, Winston Salem, North Carolina, United States of America; 8 Department of Radiation Oncology, Wake Forest University School of Medicine, Winston Salem, North Carolina, United States of America; Katholieke Universiteit Leuven, Belgium

## Abstract

Medications that can mitigate against radiation injury are limited. In this study, we investigated the ability of recombinant human growth hormone (rhGH) to mitigate against radiation injury in mice and nonhuman primates. BALB/c mice were irradiated with 7.5 Gy and treated post-irradiation with rhGH intravenously at a once daily dose of 20 µg/dose for 35 days. rhGH protected 17 out of 28 mice (60.7%) from lethal irradiation while only 3 out of 28 mice (10.7%) survived in the saline control group. A shorter course of 5 days of rhGH post-irradiation produced similar results. Compared with the saline control group, treatment with rhGH on irradiated BALB/c mice significantly accelerated overall hematopoietic recovery. Specifically, the recovery of total white cells, CD4 and CD8 T cell subsets, B cells, NK cells and especially platelets post radiation exposure were significantly accelerated in the rhGH-treated mice. Moreover, treatment with rhGH increased the frequency of hematopoietic stem/progenitor cells as measured by flow cytometry and colony forming unit assays in bone marrow harvested at day 14 after irradiation, suggesting the effects of rhGH are at the hematopoietic stem/progenitor level. rhGH mediated the hematopoietic effects primarily through their niches. Similar data with rhGH were also observed following 2 Gy sublethal irradiation of nonhuman primates. Our data demonstrate that rhGH promotes hematopoietic engraftment and immune recovery post the exposure of ionizing radiation and mitigates against the mortality from lethal irradiation even when administered after exposure.

## Introduction

The misuse of ionizing radiation or nuclear devices as weapons of terrorism has been recognized as a major public health threat [1], [2]. In the event of a nuclear detonation, terrorist radiological (e.g., “dirty”) bomb, or attack on a nuclear power plant, casualties may be generated well outside the periphery of the lethal zone. Depending on the type of nuclear device, these casualties may range from trivial biological exposures (nonetheless causing severe anxiety) to acute high-dose exposures that result in the development of severe radiation sickness and death. Typically, individuals exposed to ionizing radiation doses in the range of 0.7 to 4 Gy will develop symptoms that are secondary to hematopoietic and immune damage [3]. At exposures approximating 4 Gy, it is estimated that 50% of individuals will die within 60 days unless there is medical intervention [1], [3]. The majority of deaths that occur from exposures of 4–10 Gy also result, in a large part, from the sequelae of hematopoietic and immune failure (bleeding and infections). In addition, even at levels of radiation exposure significantly lower than those needed to cause symptoms of radiation sickness, there are alterations of the immune system so that the virulence and infectivity of biological agents (bacteria, viruses and fungi) are dramatically increased [4], [5]. A compromised immune system exacerbates the effects of infectious agents, including other biological pathogens such as anthrax, and may preclude the use of vaccines. Unfortunately, therapeutic agents capable of promoting or accelerating the recovery of the hematopoietic and/or immune compartments following radiation injury are limited [1], [2], [3].

The potential relationship between the neuroendocrine system and hematopoiesis has been postulated for many years [6]. Growth hormone, which is produced by the anterior pituitary, has been demonstrated to have a stimulatory role in erythropoiesis [7], [8] and granulopoiesis [9] either through direct effects or indirectly via the action of insulin-like growth factor 1 (IGF-1) [7], [8]. Growth hormone also stimulates lymphocyte production in rodents and growth hormone replacement in hypophysectomized animals has been associated with recovery of thymic function [10], [11]. These biologic features, along with its demonstrated safety profile of recombinant human growth hormone (rhGH) in humans, make rhGH an attractive candidate for use in the treatment of victims of ionizing radiation exposure where one's hematopoietic and immune systems can be rapidly and severely depleted.

In this study, we investigated the utility of rhGH following lethal and sub-lethal irradiation and its effect on the reconstitution of hematologic and immune systems using both mouse and nonhuman primate models. The results indicate that rhGH enhances both hematologic engraftment and immune recovery and mitigates the mortality from lethal and non-lethal irradiation. These beneficial effects are a result of enhanced hematopoiesis after treatment with rhGH. rhGH augments hematopoiesis mainly through impacting hematopoietic niches.

## Results

### rhGH mitigates against lethal irradiation

**Figure 1:**
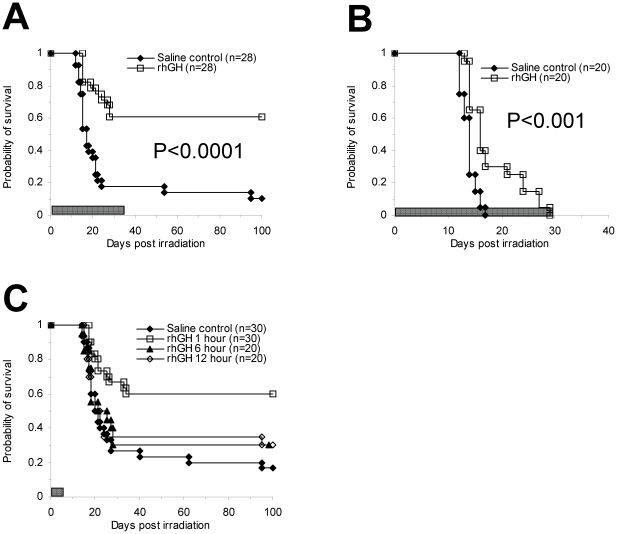
rhGH mitigates against lethal irradiation. BALB/c mice were irradiated with 7.5 Gy (all except B) or 8.5 Gy (B) and treated with rhGH i.v. daily at a dose of 20 µg/dose/day for 5 or 35 days. Unless where indicated, rhGH was given within one hour post irradiation. Survival was monitored daily. The bars represent the period of the time that the animals were treated. The data were from the combined results from 2–3 experiments. Each experiment had similar design (n = 8–10 animals per group) and results. (A) Treated for 35 days; (B) 8.5 Gy irradiation and treated daily until death; (C) Treated for 5 days and time of the first dose. P<0.05, 1 hr vs. other groups. rhGH stands for recombinant human growth hormone.

We first tested the ability of rhGH to mitigate against lethal irradiation in mice. BALB/c mice were whole-body irradiated with 7.5 Gy and treated with rhGH intravenously once daily at a dose of 20 µg/dose/day for 35 days starting within one hour post radiation exposure. As shown in Fig. 1A, 60.7% of the mice treated with rhGH survived more than 100 days post radiation exposure while only 10.7% of the mice in the control group survived, demonstrating that rhGH possesses significant radioprotective effect. This effect was still detectable when the radiation dose was increased to 8.5 Gy although all the animals ultimately died (Fig. 1B). To further characterize the radioprotective effects of rhGH, we sought to determine whether we could inject rhGH less frequently. When we shortened the treatment time from 35 days to 5 days, the radioprotective effect was still significant (Fig. 1C, 100-day survival: 60% vs. 20%).

Because victims may not be able to receive medical treatment until several hours to days following radiation exposure, it would be useful to determine how long we can wait before the administration of the first dose of rhGH. Administration of the first dose of rhGH after 6 and 12 hours demonstrated a trend to improved survival but did not achieve statistical significance (Fig. 1C).

The mice used for the above experiments were 8–12 weeks old BALB/c mice. To ascertain that the effects of rhGH were not strain specific, we repeated the experiment with C57BL/6 mice. C57BL/6 mice were irradiated with 9 Gy and treated with rhGH (20 µg/dose/day, i.v.) once a day for 5 days starting within one hour post radiation exposure. As demonstrated in Fig. S1, similar to the data obtained from BALB/c mice, rhGH was also able to mitigate against lethal irradiation in C57BL/6 mice.

It is important to point out that all the irradiated mice survived more than 100 days remained healthy for more than one year. All animals appeared to be normal. When tested more than one year post irradiation, the hematologic and immune parameters remained within the normal range and no tumors were found in all survivors at autopsies.

### rhGH facilitates hematologic recovery post radiation exposure

**Figure 2:**
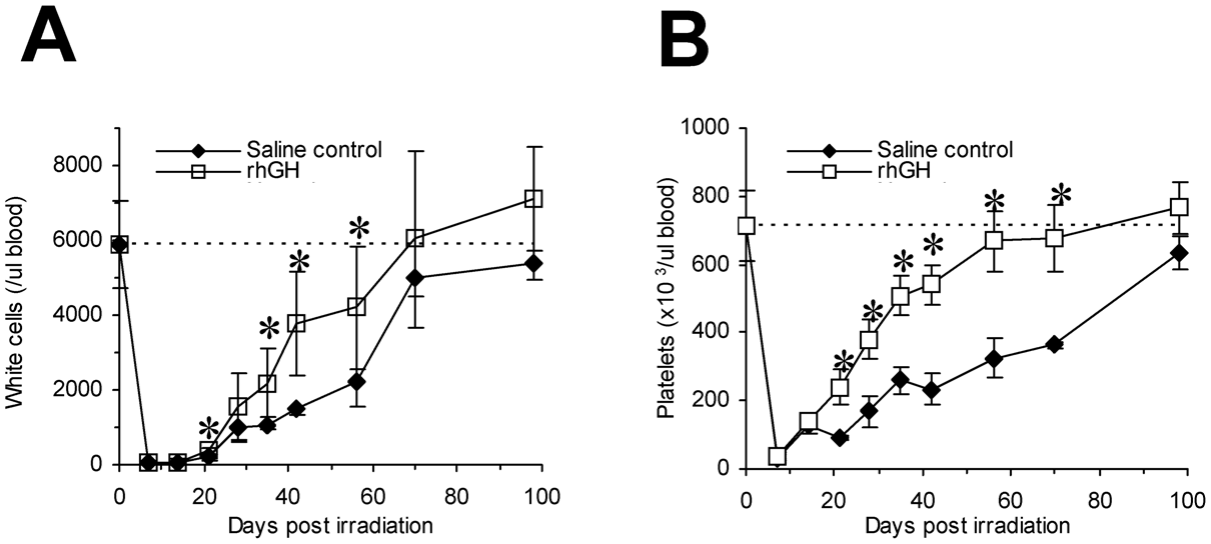
rhGH facilitates hematological recovery post irradiation. BALB/c mice were irradiated with 7.5 Gy and treated with rhGH for 35 days. White counts were determined by flow cytometer using CD45 antibody. Platelet counts were determined by hemacytometer. The dash line represents the pre-irradiation level. Each group contained 10 animals. (A) White cells; (B) Platelets. ^*^P<0.05, rhGH vs. saline control as analyzed by Student's t-test; P<0.01, rhGH vs. saline control as analyzed by repeated measures ANOVA. rhGH stands for recombinant human growth hormone.

Hematopoietic cells are amongst the most sensitive cell types to ionizing radiation and the mice often die from the sequelae of hematopoietic and immune failure (bleeding and infections) [1], [3]. To understand how rhGH mitigates against lethal irradiation, we monitored the recovery of white cells and platelets in peripheral blood following irradiation with 7.5 Gy. As demonstrated in Fig. 2A, treatment with rhGH significantly accelerated the recovery of total white blood cells. The difference in white counts was first detected at day +21 and lasted until day +56. The white cell counts in both groups recovered back to normal or near normal at day +70 and completely normal by day +100. Similar results were observed with platelets (Fig. 2B). Platelet counts in the rhGH-treated group recovered back to normal by day +56 while the platelet counts in the saline control group did not recover to normal levels until day +100.

### rhGH enhances immune recovery post radiation exposure

**Figure 3:**
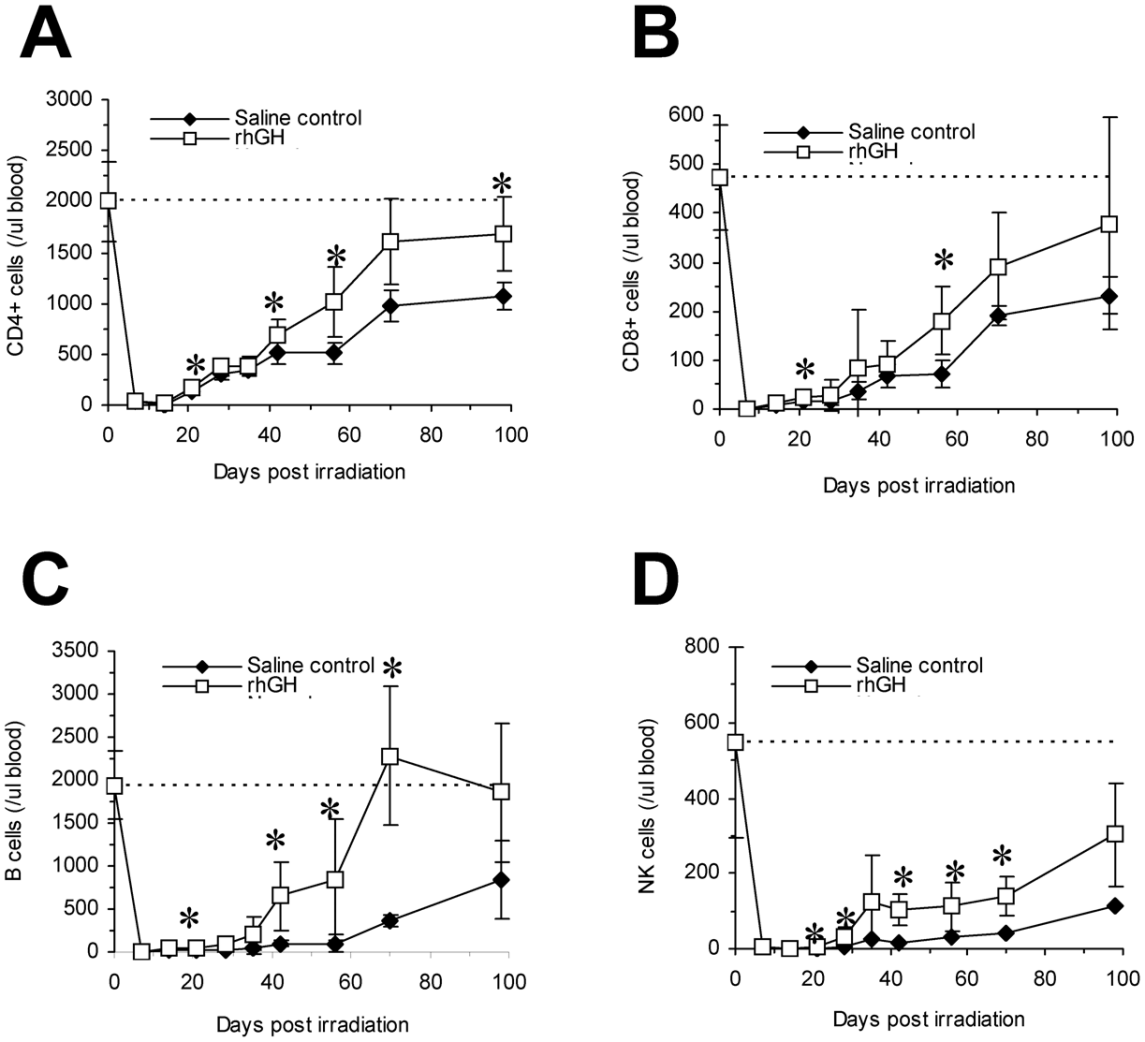
rhGH enhances immune recovery post irradiation. BALB/c mice were irradiated with 7.5 Gy and treated with rhGH for 35 days. Phenotypic immune recovery was monitored in peripheral blood by flow cytometry. The dash line represents the pre-irradiation level. Each group contained 10 animals. (A) CD4^+^ T cells; (B) CD8^+^ T cells; (C) B cells; (D) NK cells. *P<0.05, rhGH vs. saline control as analyzed by Student's t-test; P<0.01, rhGH vs. saline control for CD4+ and B cells as analyzed by repeated measures ANOVA. rhGH stands for recombinant human growth hormone.

Infections are one of the most frequent consequences following significant radiation exposure and are caused by the weakened immune system [3], [5]. Since rhGH has previously been demonstrated by our group and others to be able to facilitate immune recovery post hematopoietic stem cells transplantation and immunosuppressive therapy [10], [11], [12], we sought to determine whether rhGH can elicit the same effect post radiation exposure on the residual host cells. Thus, we monitored the kinetics of T, B, and NK cell recovery in peripheral blood in the irradiated (7.5 Gy) mice treated with rhGH. As demonstrated in Fig. 3, rhGH facilitated the recovery of CD4^+^ T cells and B cells (B220^+^CD3^−^CD49b^−^). Even though there were clear treads toward the increase of CD8+ T cell and NK cell (CD3^−^CD49b^+^) counts in the rhGH-treated mice compared with the control mice, the differences were not statistically different as analyzed by repeated measures analysis of varience (ANOVA). When analyzed by Student's t-test, significant differences were observed in multiple time points in both CD8^+^ T cell and NK cell (CD3^−^CD49b^+^) counts as indicated in Fig. 3. CD4^+^ T cell, CD8^+^ T cell, and B cell counts in rhGH-treated mice recovered back to normal or near normal by day +100. By contract, all lymphocyte counts were only half or less than half of the normal levels in the saline control group at day +100.

### rhGH promotes the recovery of hematopoietic stem/progenitor cells post radiation exposure

**Figure 4:**
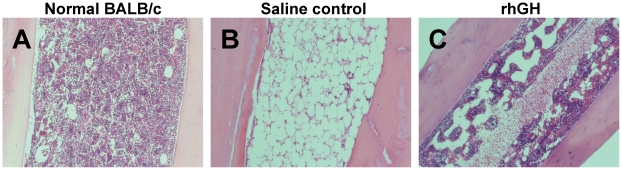
Histological analyses. BALB/c mice were irradiated with 7.5 Gy and treated with rhGH i.v. daily at a dose of 20 µg/dose/day. Mice were sacrificed on day +14 and tibia was stained with hematoxylin and eosin and analyzed. The magnification was 12.6×. (A) Normal BALB/c mice; (B) Saline control; (C) rhGH. rhGH stands for recombinant human growth hormone.

Growth hormone has been demonstrated to have a stimulatory role in erythropoiesis [7], [8] and granulopoiesis [9]. We have also demonstrated that the number of hematopoietic stem/progenitor cells positively correlates with the speed of hematological and immune recovery [13]. Based on these previously published data, we hypothesized that rhGH facilitates hematologic and immune recovery following irradiation by promoting hematopoiesis. To test this hypothesis, we measured the recovery of hematopoietic stem/progenitor cells post irradiation by flow cytometry and colony forming unit assays. BALB/c mice were irradiated (7.5 Gy) and treated with rhGH daily starting within one hour post irradiation. At day +14, all mice were sacrificed and the bone marrow was harvested in order to quantify the number of hematopoietic stem and progenitor cells. We first measured the content of hematopoietic progenitor cells by colony forming unit assays. As demonstrated in Table 1, the bone marrow from the rhGH-treated mice had significant higher numbers of CFU-GM and BFU-E and CFU-GEMM compared with the saline control mice did. The numbers of BFU-E and CFU-GEMM were even higher than the normal level (Table 1). Interestingly, the number of nucleated cells per colony was also significantly higher in the rhGH-treated group than that in the saline control group, suggesting that the ability of progenitors to generate the progenies is also enhanced after rhGH treatment. Consistent with the data from the colony forming unit assays, the frequency of c-Kit^+^Sca-1^+^Lin^−^ (KSL) cells, which are highly enriched for both long and short term stem cells, was higher in the rhGH-treated group than that in the saline control group. Further testing of long term hematopoietic stem cells (CD150^+^CD48^−^CD244^−^) [14] in bone marrow demonstrated that the frequency of these cells was also increased in rhGH-treated mice compared with that in the saline control mice. Histological analyses at day 14 post irradiation (Fig. 4) demonstrated that the treatment with rhGH significantly increased the cellularity in bone marrow as compared with the saline control, further confirming that rhGH enhances hematopoietic recovery post radiation exposure.

**Table 1:** Effect of rhGH on hematopoiesis.

Groups	CD150^+^ CD48^−^ CD244^−^	KSL cells	CFU-GM	BFU-E	CFU-GEMM	Cells (×10^4^) per colony
	(/10^5^ bone marrow cells)					
Normal BALB/c	ND	80.1±24.3	45.4±6.3	8.4±0.9	4.9±1.2	7.1±0.6
Saline control	0.6±0.6	11.8±5.1	14.3±2.4	6.1±2.3	2.6±0.5	4.5±1.3
rhGH	4.1±2.6^a^	29.3±16.8a,b	36.8±10.6^a^	11.2±2.2a,b	7.6±1.8a,b	7.7±0.7^a^

BALB/c mice were irradiated with 7.5 Gy and treated with rhGH for 5 days. Mice were sacrificed and bone marrow was harvested at day +14 post irradiation. Frequencies of CD150^+^CD48^−^CD244^−^ and KSL cells were determined by flow cytometry. CFUs were determined after 12 days in culture. Each group contains 4–10 animals. This is a representative of three similar experiments. rhGH stands for recombinant human growth hormone; KSL cells, cells expressing c-Kit^+^Sca-1^+^Linage^−/low^ phenotype; CFU-GM, colony forming unit-granulocyte & monocyte; BFU-E, burst forming unit-erythrocyte; CFU-GEMM, colony forming unit-granulocyte, erythrocyte, monocyte, and megakaryocyte.

a P<0.05, saline control vs. rhGH;

b P<0.05,. rhGH vs. normal BALB/c.

### Effects of rhGH on apoptotic and mitotic death of hematopoietic stem/progenitor cells post radiation exposure

**Figure 5:**
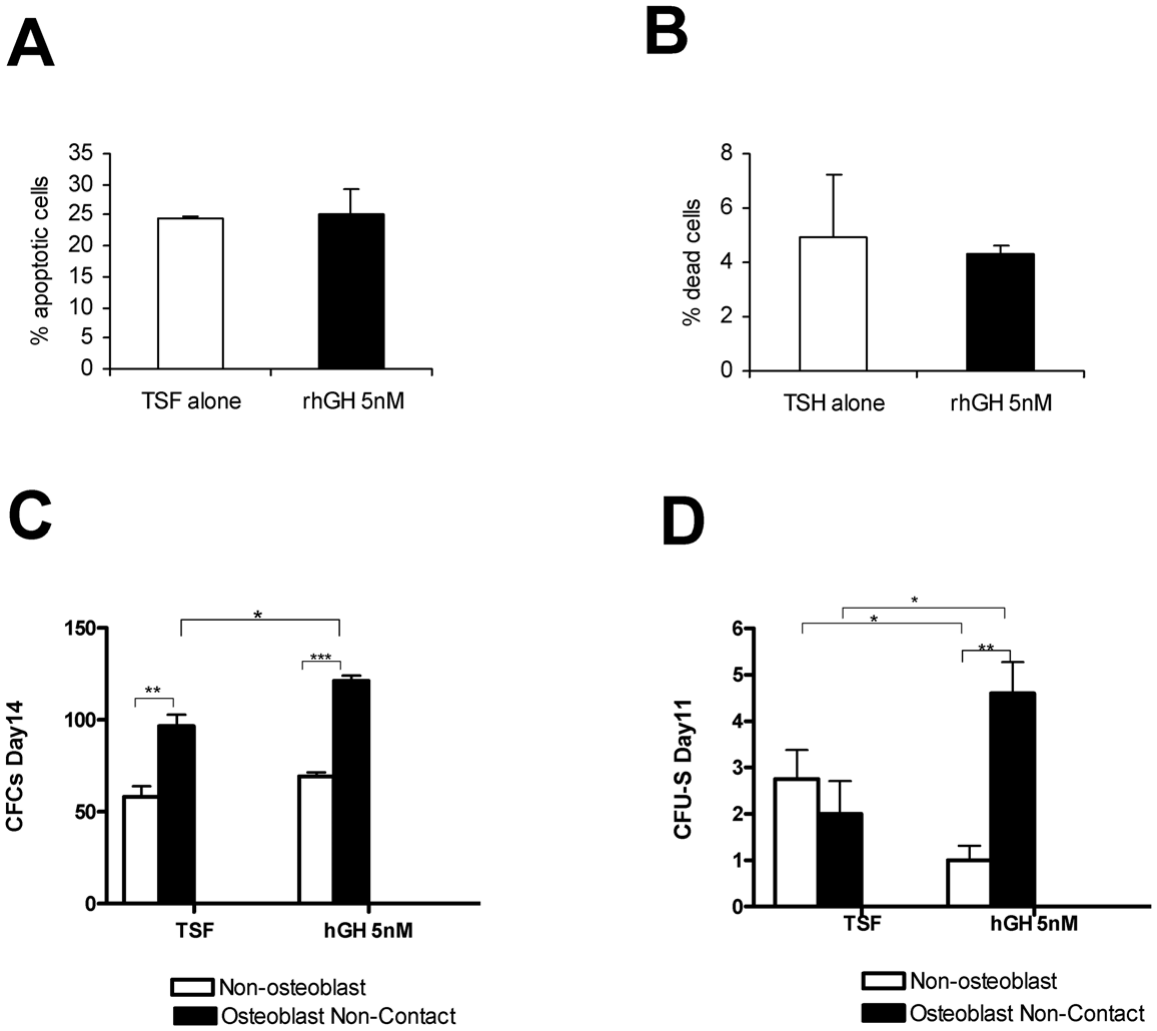
In vitro effecs of rhGH on hematopoietic stem/progenitor cells. Irradiated (3 Gy [C,D] or 4 Gy [A,B]) c-Kit+Lin-Sca-1+ cells from C57BL/6 mice were cultured with thrombopoietin, stem cell factor, Fl3 ligand and various concentration of recombinant human growth hormone. In some experiments shown in C and D, osteoblasts were added to the culture. After 24 hours in culture, percentages of apoptotic (A) and dead (B) cells among c-Kit^+^Lin^−^Sca-1^+^ hematopoietic stem/progenitor cells were determined by flow cytometry. Similar results were obtained at 48 and 72 hours. In the experiments shown in C and D, the cells were harvested after 7 days in culture. The frequencies of hematopoietic stem and progenitor cells were then determined by in vitro colony forming unit assay (C) and in vivo CFU-S assay (D) respectively. ^*^P<0.05, ^**^P<0.01, ^***^P<0.001. rhGH stands for recombinant human growth hormone; TSF, thrombopoietin, stem cell factor, Fl3 ligand, CFU-S, colony forming unit-spleen; CFC, colony forming cell.

Cells die either through apoptosis or mitotic death following irradiation. In order to determine how rhGH protects hematopoietic stem/progenitor cells post radiation exposure, we irradiated purified KLS cells from C57BL/6 bone marrow at 4 Gy and cultured them with 5 nM of rhGH (this concentration was chosen based on the published data [7], [8], [9] and our preliminary testing) in the presence of thrombopoietin, stem cell factor, and Flt3 ligand. The percentages of apoptotic and dead cells were then determined by flow cytometry after stained with 7-AAD and annexin V at various time points post radiation exposure. As demonstrated in Fig. 5A, rhGH did not protect hematopoietic stem/progenitor cells from undergoing apoptosis when measured 24 hours post treatment. No significant effects on cell death were observed after treatment with rhGH when measured at 24 hours post radiation exposure (Fig. 5B). Similar data were observed when measured at 48 and 72 hours post irradiation (data not shown).

### In vitro effect of rhGH on irradiated hematopoietic stem and progenitor cells

In order to further understand how rhGH promoted hematopoietic recovery post irradiation, we studied the effect of rhGH on irradiated hematopoietic stem and progenitor cells using in vitro assays. Purified KSL cells from C57BL/6 bone marrow were irradiated with 3 Gy and cultured with 5 nM of rhGH in the presence of thrombopoietin, stem cell factor, and Flt3 ligand (TSF). After 7 days in culture, the cells were harvested and the numbers of CFCs and CFU-S were determined. As demonstrated in Fig. 5C, rhGH did not seem to have an effect on the number of CFCs, which measures the frequency of hematopoietic progenitor cells. Surprisingly, rhGH seemed to decrease the number of day 11 CFU-S (Fig. 5D), which is a measurement for long-term hematopoietic stem cell content. These data suggest that rhGH has limited direct effects on irradiated hematopoietic progenitor cells and has negative direct effects on irradiated long-term hematopoietic stem cells.

To determine whether rhGH can augment hematopoiesis indirectly through other cell types, we added osteoblasts in the cultures. Osteoblasts have been recently demonstrated to be a critical component of the hematopoietic stem cell niche [15], [16], [17]. The results in Fig. 5C, D demonstrated that osteoblasts independently increased CFCs but not the number of day 11 CFU-S. In the presence of osteoblasts, rhGH significantly increased the number of CFU-S and trended to increase CFCs compared with TSF alone. All the effects of osteoblast are mediated by soluble factors because the osteoblasts had no direct contact with hematopoietic stem/progenitor cells. These data indicate that rhGH may augment hematopoietic stem/progenitor cell activity through osteoblasts.

### rhGH increases the plasma level of IGF-1

**Figure 6:**
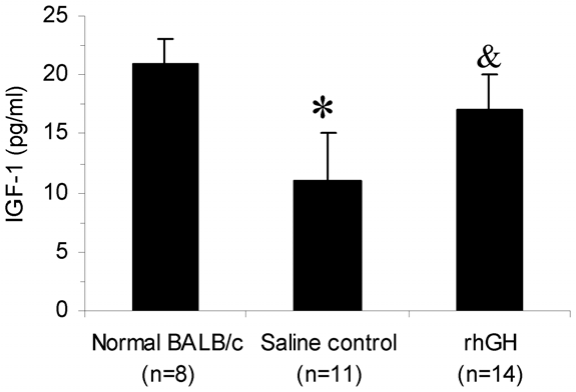
rhGH increases the plasma level of IGF-1. BALB/c mice were irradiated with 7.5 Gy and treated with rhGH for 5 days. Plasma IGF-1 levels were determined by ELISA. ^*^P<0.0001, saline control vs. other groups; ^&^P = 0.01, rhGH vs. normal BALB/c. rhGH stands for recombinant human growth hormone; IGF-1, insulin-like growth factor.

It was previously demonstrated that growth hormone mediates at least some of its effects on hematopoiesis through production of IGF-1 [9]. To investigate whether it is the case in our model, we measured the plasma level of IGF-1 at day +14 post irradiation. IGF-1 levels were decreased in all irradiated mice compared with normal BALB/c mice (Fig. 6). After treated with rhGH, IGF-1 levels were significantly increased compared with the saline control group, demonstrating that the treatment with rhGH does increase the blood level of IGF-1.

### Effect of rhGH on hematologic and immune recovery post sublethal irradiation in nonhuman primates

**Figure 7:**
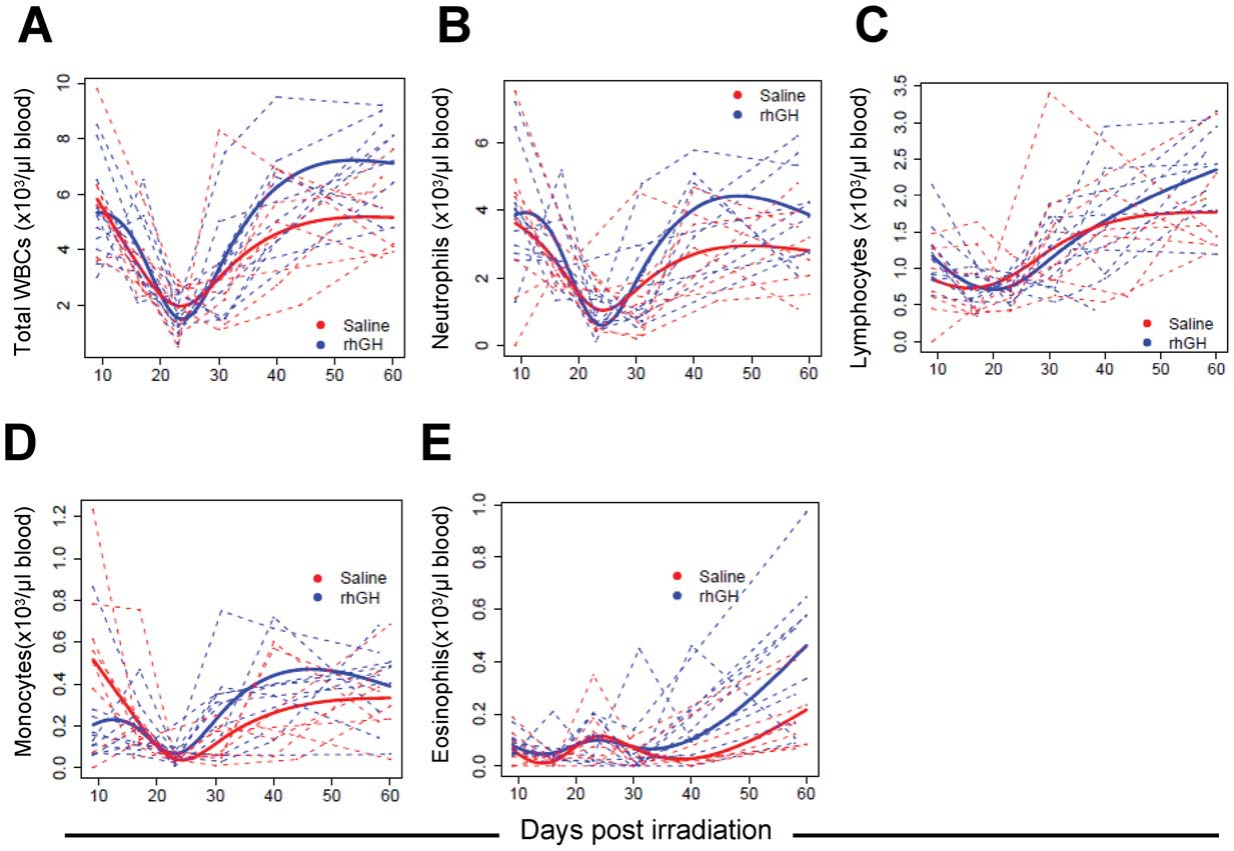
rhGH accelerates white blood cell recovery post sublethal irradiation in nonhuman primates. After irradiated with sublethal whole body irradiation (2 Gy), cynomolgus monkeys were treated with rhGH at a once daily dose of 5 µg/kg for 30 days. Peripheral blood counts were measured overtime. The data were combined from two similar experiments. There were 8 animals (n = 4 for both study #1 & #2) in the saline control group and 11 animals (n = 6 in study #1, n = 5 in study #2) in the rhGH-treated group. The data were analyzed by using a regression model. Data from individual animals (dash line) as well as fitted lines (solid lines) were shown. (A) Total white blood cells. P<0.05. (B) Neutrophils. (C) Lymphocytes. (D) Monocytes. P<0.01. (E) Eosinophils. P<0.05.

The data presented above convincingly demonstrate that rhGH enhances both hematologic and immune recovery post radiation exposure in mice. Since similar clinical studies can not be ethically conducted in humans, demonstration of similar results in a second different species other than rodents would be needed before it can be used as a medical countermeasure for radiation injury in humans [18]. Because of the high degree of genetic and physiologic similarity of nonhuman primates to human beings [19], [20], cynomolgus monkeys were chosen as a second animal model to test the efficacy of rhGH. Cynomolgus monkeys were irradiated with 2 Gy whole-body and treated with rhGH at a once daily dose of 5 µg/kg for 30 days. The control animals were irradiated and injected with saline. Peripheral blood cell counts were assayed daily for the first 5 days, then 3 times weekly for the remainder of a 30-day period the first month, every other week in the second month, and once a month thereafter. As shown in Fig. 7, time trend analyses of the data using a regression model demonstrated that rhGH accelerated the recovery of total white blood cells (P<0.05), monocytes (P<0.01), and eosinophils (P<0.05) and trended to a faster recovery of neutrophils and lymphocytes compared with saline control. Even though there were clear treads toward increased neutrophils and lymphocytes in the rhGH treated cynomolgus monkeys (Fig. 7), the differences were not significant, probably due to small sample sizes. Nevertheless, further analyses using Student's t-test demonstrated statistical differences between rhGH- treated and saline control groups in total white blood cells (7170±1600 vs. 5010±1450, P = 0.01), lymphocytes (2440±650 vs. 1790±650, P<0.05), and eosinophils (490±330 vs. 200±150, P<0.05) at week +9 and total white blood cells (9940±2200 vs. 6100±590, P = 0.01), neutrophils (6180±2220vs. 3080±1170, P<0.05), and monocytes (600±200 vs. 310±60, P<0.05) at week +18. Taken together, significant increased numbers of total white blood cells and all major white cell subsets were demonstrated in rhGH treated nonhuman primates after sublethal irradiation. No effects of rhGH on red blood cell, hematocrit, hemaglobin, and platelet recovery were detected (data not shown). The differences in all cell counts resolved after 22 weeks post irradiation. Similar to the mouse data, these data demonstrate that rhGH is able to enhance the recovery of total white cells, neutrophils, lymphocytes, monocytes, and eosinophils post sublethal radiation injury in nonhuman primates, suggesting the recovery of both innate and adaptive immunity are enhanced after rhGH treatment.

## Discussion

Previously published data have demonstrated that rhGH enhances hematologic engraftment and immune recovery post hematopoietic stem cell transplantation [12], [21]. In this report, we have further demonstrated that rhGH also has significant radioprotective effects against lethal irradiation in two different strains of mice when administrated post radiation exposure. This effect is a result from the positive effects of rhGH on hematopoietic recovery. In addition, rhGH enhances immune reconstitution post radiation exposure. We have further confirmed the beneficial effects of rhGH in post-irradiation white cell recovery using a nonhuman primate model.

Different cells and tissues have different sensitivities to ionizing radiation [2], [22]. Hematopoietic cells are among the cell types that are most sensitive to radiation[2]. In fact, the majority of deaths that occur from exposures of 4–10 Gy result, in a large part, from the sequelae of hematopoietic and immune failure (bleeding and infections) [3]. Even though a number of agents such as thiol compounds [23], [24],interleukin (IL)-1 [23], [24], [25], tumor necrosis factor α [25], [26], stem cell factor [26], [27], IL-12 [28], basic fibroblast growth factor [29], flt3 ligand [30], [31], CBLB502 [32], granulocyte colony stimulating factor (G-CSF) [23], [33], [34], granulocyte & macrophage colony stimulating factor (GM-CSF) [35], and combinations of cytokines and growth factors [36] have been shown to be able to protect against lethal irradiation, only a few of them such as CBLB502 [32], G-CSF [23], [33], [34], GM-CSF [35] are effective when administered post radiation exposure. None of them have been approved by the Food and Drug Administration for the management of radiation-induced marrow aplasia so far. We chose to study rHGH as a radiation mitigator because it is an approved drug, available in the market and thus may be easier to be developed as a medical countermeasure for radiation injury in humans. In addition, rhGH can also be used in combination with G-CSF or GM-CSF because rhGH can also facilitate immune recovery (Fig. 4) while G-CSF [37] and GM-CSF [38] can not.

Even though both G-CSF and rhGH are effective in protection against lethal radiation, the window of radiation doses for which they have an effect is narrow. Treatment with rhGH leads to long term survival of more than 60% of the BALB/c mice irradiated with 7.5 Gy (Fig. 1A) while all the BALB/c mice irradiated with 8.5 Gy survive longer after treatment with rhGH, but die within 30 days (Fig. 1B). Similarly, G-CSF is able to rescue about half of the BDF1 mice irradiated with 9.5 Gy [33] but G-CSF is not effective anymore after the radiation dose is increased to 10.5 Gy [33]. These observations suggest that, similar to G-CSF, the use of rhGH will be restricted to a narrow window of radiation doses when used as a sole agent to mitigate lethal irradiation and that the effects are primarily on the hematopoietic system. While the mice were kept in a specific pathogen free facility during the studies, mortality could be a result of infections. Yet we could not detect bacteria in blood cultures performed on moribund mice at various timepoints post irradiation (data not shown), suggesting that death from infections was unlikely. Another target organ of irradiation for acute mortality at the dose of 7.5 Gy could be intestine [22]. However, the level of citruline, a reliable indicator of small intestine injury [39], recovered back to normal quickly in both control and treated groups (Table S1), suggesting that gut injury is not a primary cause of death at this dose of radiation. Another issue is how long we can delay the treatment after irradiation. This practical aspect is important because radiation victims may not be able to receive treatment until hours or days post exposure. For example, G-CSF is not very effective when given 24 hours post exposure [34]. Similarly, the effect of rhGH is decreased when the first dose is delayed until 6 and 12 hours post irradiation (Fig. 1C). These observations indicate that the radiation victims need to be treated as soon as possible after exposure at least for the acute effects of rhGH on hematopoiesis. It is of note that rhGH could be used later for its effects on the immune system [40].

At the radiation dose of 7.5 Gy, death is usually caused by hematological failure [3]. Indeed, both white cell and platelet counts drop to a very low level (Fig. 2) and the bone marrow was essentially empty (Fig. 4). Treatment with rhGH significantly accelerates the recovery of both white cell and platelet counts (Fig. 2), suggesting that the effects of rhGH may be at the hematopoietic stem/progenitor level. Histological analysis (Fig. 4) supports this notion as the cellularity in the bone marrow of rhGH-treated mice is dramatically increased comparing with that in the saline control mice. Measurement of CFCs in the bone marrow at day +14 (Table 1) conclusively demonstrate the contents of all lineages of hematopoietic progenitor cells are increased after rhGH treatment. Increase frequencies of bone marrow KSL cells as well as more primitive CD150^+^CD48^−^CD244^−^ cells [14] (Table 1) indicates that rhGH works at the stem cell level. rhGH also increases the proliferative activity of hematopoietic progenitor cells because the cells per colony are statistically increased in the mice treated with rhGH (Table 1). These observations suggest that rhGH not only promotes the recovery of hematopoietic stem and progenitor cells but also enhances the ability of progenitor cells to generate their progenies. Both pathways should contribute to the accelerated recovery of peripheral blood counts post rhGH treatment, which is crucial for the survival of radiation victims.

How does rhGH promote hematopoietic stem/progenitor recovery post irradiation? Ionizing radiation damages DNA and causes both apoptotic and mitotic death [22]. rhGH could protect hematopoietic stem/progenitor cells from apoptosis and mitotic death [41]. However, our data shown in Fig. 5A, B indicate that rhGH does not protect against either apoptosis or death of irradiated hematopoietic stem/progenitor cells. Because some of the hematopoietic stem and progenitor cells do survive even after lethal dose of irradiation [42], another possibility is that rhGH promotes the expansion of the surviving hematopoietic stem/progenitor cells directly or indirectly. But our in vitro data presented in Fig. 5C, D suggest that rhGH has limited direct effects on hemapoietic stem/progenitor cells. It is most likely that rhGH promotes the expansion of the surviving hematopoietic stem and progenitor cells indirectly through other cell types such as osteoblast in the hematopoietic niches as suggested by the data presented in Fig. 5C, D. Osteoblasts have recently been recognized as an important component of hematopoietic stem cell niche [15], [16], [17]. rhGH activates osteoblasts after binding to the growth hormone receptor expressed on osteoblasts [43]. Upon activation, osteoblasts can produce several necessary soluble and membrane-associated factors capable of regulating hematopoietic stem/progenitor cells [44], [45]. Indeed, rhGH is able to expand hematopoietic stem and progenitor cells only when osteoblasts are present (Fig. 5C, D), demonstrating that rhGH can promote irradiated hematopoietic stem/progenitor cell activity through its effects on osteoblasts. These data are consistent with the previously published data indicating rhGH requires stromal cells to stimulate erythropoiesis and granulopoiesis [7], [8], [9], [46], [47]. The fact that direct contact between osteoblasts and hematopoietic stem/progenitor cells is not required for rhGH's effects (Fig. 5C, D) suggests that the stimulating effects are mediated by soluble factor(s). It has been previously demonstrated that osteoblasts can produce several hematopoietic cytokines including G-CSF, GM-CSF, IL-6, leukemia inhibitory factor, and stromal-derived factor 1 [44], [45]. It is currently not clear whether rhGH can promote osteoblasts to secrete these hematopoietic cytokines. Because rhGH is able to induce bone formation [48], another pathway that rhGH uses to promote hematopoiesis is through increasing the number of osteoblasts. However, trabecular bone fraction (% BV/TV) as determined by quantitative microCT in tibias harvested at 14 days post irradiation is similar between the rhGH-treated and saline control groups (data not shown), suggesting that an increase of trabecular bone formation may not be responsible for the enhanced hemapoiesis after treatment with rhGH.

It was previously demonstrated that rhGH mediates at least some of its effects on hematopoiesis through production of IGF-1 [9]. In our model, treatment with rhGH significantly increases systemic level of IGF-1 (Fig. 6). Because it is known that IGF-1 can promotes hematopoiesis [49], the increased plasma level of IGF-1 likely leads to enhanced hematopoietic recovery. Indeed, it has been demonstrated that IGF-1 decreases apoptosis and promotes survival of hematopoietic progenitors in a PI3-kinase-dependent manner [50], [51]. IGF-1 activates the serine/threonine kinase Akt through PI3-kinase. This activation phosphorylates and inhibits caspase 3 resulting in decreased apoptosis [52].

We have previously demonstrated that the number of hematopoietic stem/progenitor cells directly correlates with the speed of immune reconstitution [13]. Since the number of hematopoietic stem cells is increased in the rhGH-treated mice (Table 1), it was expected that immune recovery is also accelerated in these animals as demonstrated in Fig. 3. rhGH can also stimulate the expansion of mature immune cells directly [41]. The fact that only the numbers of white cell and its subsets are increased but not those of red blood cells and platelets after treatment with rhGH in sublethal irradiated nonhuman primates (Fig. 7) does suggest this could be the case. Nevertheless, the positive effects of rhGH on immune recovery make rhGH a very attractive candidate for a medical countermeasure against radiation injury because infections are one of major causes of death [4], [5]. In fact, it was recently reported that rhGH is able to enhance thymopoiesis in HIV/AIDS patients [53]. A clinical trial has also been initiated in our group to test the efficacy of rhGH toward enhancing immune reconstitution post cord blood transplantation.

There are several theoretical concerns with the use of rhGH [6]. One of the concerns is whether rhGH promotes the autoimmune phenomena because rhGH potentiates the proliferation and cytokine responses of T cells in response to antigens. Another concern is whether rhGH promotes the growth of cancers. In order to address these concerns, we monitored the surviving mice treated with rhGH for more than one year post irradiation. All the animals appeared to be normal throughout the study. Both the body weights and hematological and immune parameters in the long term survivors were normal. No tumors were detected in any long term survivors. These observations suggest that rhGH is unlikely to promote autoimmunity and the growth of cancer in the radiation victims. Moreover, the clinical monitoring in normal individuals with short stature receiving rhGH has not demonstrated an increased risk of malignancies or autoimmune diseases [54].

In conclusion, rhGH promotes hematopoietic and immune recovery post radiation exposure and protects against lethal and sub-lethal irradiation when administered after irradiation. rhGH is an attractive candidate for mitigation of toxicity from irradiation and should be tested further as a medical countermeasure against radiation injury.

## Materials and Methods

### Ethics statement

All mouse experiments were performed under research protocols approved by the Duke University Animal Care and Use Committee and were accordance with the National Institutes of Health Guide for the Care and Use of Laboratory Animals. All procedures involving nonhuman primates were approved by the Institutional Animal Care and Use Committee of Wake Forest University, under accreditation by the Association for the Assessment and Accreditation of Laboratory Animal Care.

### Mice

BALB/c (H2^d^) and C57BL/6 (H2^b^) were purchased from The Jackson Laboratories (Bar Harbor, Maine). Eight to twelve weeks old female mice were used in this study, unless specifically noted. The mice were housed in a specific pathogen free facility throughout the study. The mice were provided with autoclaved food and acidified water. In our animal facility, sentinel mice were used to ensure that the facility is clean. Serum samples from sentinel mice were sent to the Research Animal Diagnostic Laboratory at University of Missouri (Columbia, MO) once a quarter to test for the following organisms: cilia associated respiratory bacillus, ectromelia, encephalitozoon cuniculi, epizootic diarrhea of infant mice virus, theiler's murine encephalomyelitis virus, hantaan virus, mouse pneumonitis virus, lymphocytic choriomeningitis virus, mouse adenovirus-rodent adenovirus strain 1 (FL), mouse adenovirus-rodent adenovirus strain 2 (K87), mouse cytomegalovirus, mouse hepatitis virus, minute virus of mice, murine norovirus, mycoplasma pulmonis, mouse parvovirus, mouse thymic virus, conserved, recombinant parvoviral protein (rNS1), polyoma virus, pneumonia virus of mice, reovirus type 3, and sendai virus. During the period of our study, all the results were negative.

#### rhGH

rhGH was made by Genentech (South San Francisco, CA). For the in vivo studies, rhGH was reconstituted in saline and stored at 4°C until use according to the manufacturer's instructions. rhGH was injected into mice intravenously via tail vein at a dose of 20 µg/dose/day daily for 5 or 35 days post irradiation. This dose was demonstrated to be safe, not to significantly increase body weight, and effective in a hematopoietic cell transplantation model [12]. For the in vitro studies, rhGH was diluted in tissue culture media.

### Irradiation

The mice were whole-body irradiated in a ^137^Cs gamma irradiator (J.L. Shepherd and Associates, Glendale, CA). The dose rate was between 6.3 and 6.7 Gy/minute.

### Peripheral blood cell counts

Platelets were counted manually on a hemocytometer after being diluted in 1% ammonium oxalate solution. White blood cells were counted by flow cytometer based on CD45 staining using Flow-Count fluorospheres (Beckmen Coulter, Miami, FL). Preliminary experiments indicated that the flow cytometer is suitable for counting mouse white blood cells.

### Flow cytometry

For the murine studies, fifty µl of heparinized peripheral blood was stained with monoclonal antibodies for 15 minutes at room temperature. Red cells in the stained whole blood samples were then lysed by FACS lysing solution (Becton Dickinson, San Jose, CA). Fifty µl of Flow-Count fluorospheres (Beckmen Coulter) was added before flow cytometric analysis. The stained cells were analyzed using a FACSCanto flow cytometer (BD) equipped with FACSDiva software. The flow tubes were drained in order to collect all events. The absolute counts were calculated using the following formula: Absolute count (cells/µl blood) = (Total number of cells counted/Total number of fluorospheres counted)× Flow-Count fluorosphere concentration. The antibodies and fluorescent probes used in this study included 7-aminoactinomycin D (7-AAD), fluorescein isothiocyanate (FITC)-conjugated Annexin V and anti-CD49b (DX5); phycoerythrin (PE)-conjugated anti-CD3 (145-2C11) and anti-Sca-1 (E13–161.7); PE- cyanine (Cy)7-conjugated anti-B220 (53−6.7); allophycocyanin (APC)-conjugated anti-c-Kit (2B8); APC-Cy7-conjugated anti-CD45 (30F11) and their isotype controls from BD; Tri-color-conjugated anti-B220 (RA3-6B2) and APC-conjugated anti-CD4 (RM4-5) and their isotype controls from Caltag (South San Francisco, CA).

### Colony forming unit assay

Bone marrow cells were flushed out from femurs and strained through 70 µm strainers (Becton Dickinson, Franklin Lakes, NJ). Colony forming units was measured using Methocult GF M3434 media (Stem Cell Technologies, Vancouver, Canada) in 35 mm gridded dishes assayed at day 12–14. In some of the experiments, cultures were resuspended in 15 ml of RPMI medium to dissolve the methylcellulose following colony identification and enumeration. After washing, cell counts and viability test were performed to estimate the number of cells per colony.

### Histology

Biopsies were taken from tibia and stored in buffered formalin. Specimens were then embedded in paraffin, cut into 5 µm section, and stained with hematoxylin-eosin. The stained slides were studied under light microscope. Images were acquired with an AxioCam MRc digital camera mounted on an Axiovert 200 inverted microscope (Carl Zeiss Microimaging, Thornwood, NY). A-Plan 10x/0.25 and LD-Plan NEOFLUAR 20X/0.4 and 40X/0.6 objectives were used. Images were recorded using AxioVision Rel. 4.5 software (Carl Zeiss Microimaging). The original, unmodified pictures were used in this paper.

### Isolation of hematopoietic stem cells

This procedure has been described previously with minor modifications [13]. Whole bone marrow was collected from bilateral femurs and tibias of 8–10 week old animals by flushing with cold Dulbecco's phosphate buffered saline (Invitrogen, Carlsbad, CA), containing 10% heat-inactivated fetal bovine serum (Hyclone, Logan, UT) and 100 U/ml penicillin and 100 ug/ml streptomycin (Invitrogen). Bone marrow mononuclear cells were then isolated via density centrifugation, washed twice. Lineage-marker negative (Lin^−^) cells were then enriched via magnetic column purification using the mouse lineage cell depletion kit (Miltenyi Biotec, Auburn, CA), containing antibodies to CD5, B220, CD11b, Ly-6G/C, 4–4, and Ter-119, according to the manufacturer's recommended protocol. Multiparameter flow cytometry was conducted to isolate purified HSC subsets. Lin^−^ cells were stained with FITC-conjugated anti-Sca-1, PE-conjugated anti-c-Kit antibodies (BD), or the appropriate isotype controls. c-kit^+^sca-1^+^lin^−^ (KSL) cells were sorted in a BD FACSVantage SE flow cytometer, using FACSDiva software (Becton Dickinson). Dead cells stained with 7-AAD were excluded from analysis and sorting.

### Osteoblast cell line

MC3T3E1 clone 4 cells (ATCC, Manassas, VA) were plated at a density of 7,000 cells per well in 24-well plates in alphaMEM+10%Fetal Bovine Serum. Ascorbic acid was added to these cultures at a concentration of 50 µg/ml. The cells grew in ascorbic acid for a week, at which point the cells were washed with 1× PBS and resuspended in alphaMEM+10%FBS to prepare for co-cultures.

### In vitro culture of bone marrow stem cells

Once sorted, the murine bone marrow KSL cells were irradiated in vitro using a Cs-137 irradiator. The irradiated KSL cells were then seeded at 3,770 per well in 24-well plates (Becton Dickinson) containing 1 ml/well culture medium (IMDM with 10% heat-inactivated fetal bovine serum (Hyclone, Logan, UT) and 100 U/ml penicillin and 100 ug/ml streptomycin (Invitrogen), 20 ng/ml stem cell factor (SCF), 50 ng/ml mouse fms-like tyrosine kinase-3 (Flt-3) ligand, and 20 ng/ml mouse thrombopoietin (TSF; R&D Systems, Minneapolis, MN) with or without 7,000 osteoblastic MC3T3E1 clone 4 cells and non-contact conditions using 0.4 um transwell inserts (Corning, Lowell MA). The concentration of rhGH added to the culture was 5 nM. This concentration was chosen based on the published data [7], [8], [9] and our own preliminary experiments. Cells were cultured at 37°C, 5% CO2, 100% humidity and harvested after 7 days in culture to measure colony forming cells (CFC) and colony forming units-spleen (CFU-S). For the CFU-S assay, cells were quantified by manual hemacytometer and 20,000 live cells were injected intravenously into 9.5 Gy lethally irradiated C57BL/6 mice (5 per condition). Spleens were collected on day 11 and colonies were visualized after organ fixation using Bouin's fixative solution (RICCA Chemical Company).

### Mouse IGF-1 level

Mouse IGF-1 level was determined by enzyme-link immunosorbent assay (ELISA) using a kit purchased from Signosis (Sunnyvale, CA). Test samples and controls were added to microtiter wells coated with goat anti-mouse IGF-1 antibody. After incubation and washes, biotin labeled goat anti mouse IGF-1 antibody was added to the wells. Test samples and controls react simultaneously with the two antibodies, resulting in the IGF-1 molecules being sandwiched between the solid phase and enzyme-linked antibodies. After incubation, unbound antibodies are removed. Streptavidin conjugated to horseradish peroxidase (HRP) followed by HRP substrate are added for detection. The concentration of IGF-1 is directly proportional to the color intensity of the test sample. Absorbance is measured spectrophotometrically at 450 nm using a MRX microplate reader (Dynex Technologies, Chantilly, VA).

### Nonhuman primate studies

Adult male cynomolgus macaques (Macaca fascicularis) were purchased from the Institut Pertanian Bogor (Bogor, Indonesia), and housed socially in indoor pens at the Wake Forest University Primate Center. All animals were determined to be in good health by physical examination, and determined to be free of antibodies to simian retroviruses. Irradiations were performed using a Varian 2100 EX dual energy linear accelerator. The single fraction whole body dose of 2 Gy was given using 6 MV x rays at a dose rate of 0.69 Gy/min, using a pair of left and right lateral fields at extended distance to deliver one-half the dose (1 Gy per field) to each animal's sagittal midline. Once established, animal position was maintained for each lateral field. Anatomic measurements, phantom studies and ionization chamber measurements were used to establish the irradiation geometry and dose delivery parameters.

Clinical evaluations of macaques were done twice daily using a modification of the Children's Clinical Oncology Group toxicity criteria to assess animals for signs of illness [55]. Starting on the day of irradiation, treated animals were given a once daily subcutaneous injection of rhGH (5 ug/kg/day), into the flank; control animals were given an injection of 0.9% saline solution. This continued for 30 days after irradiation. Blood counts (hematocrit, red blood cells, red blood cell indices, total and differential leukocyte counts, hemoglobin, and platelets), serum chemistry panels (blood urea nitrogen, creatinine, total protein, albumin, bilirubin, alanine aminotransferase, aspartate aminotransferase, gamma-glutamyltransferase, potassium, sodium, chloride, and total CO2) were assayed daily (Antech Diagnostics) for the first 5 days, then 3 times weekly for the remainder of a 30-day period. Animals were sedated with ketamine for blood collection. All animals were given parenteral prophylactic antibiotic treatment, consisting of enrofloxacin (5 mg/kg) continuing until the white blood cell count returned to the normal range. Supportive fluid therapy, analgesics (ketoprofen), and symptomatic care were given as needed, based on clinical pathology abnormalities and clinical signs.

### Statistical analysis

Observed measurements were summarized as mean±SD. Comparison of cell counts, frequency of stem/progenitor cells, CFU-S, and CFCs between groups was performed by Student's t-test (two groups) or analysis of variance (ANOVA) (>two groups). When a difference was detected by ANOVA among the groups, Fisher's protected least significant difference test was used to determine the significance of pairwise differences. The cell count data shown in Fig. 2 & 3 were further analyzed by repeated measures ANOVA. Survival data were analyzed by log rank test. All statistical analyses for the mouse studies were performed using StatView software (SAS institute, Cary, NC) or Microsoft Excel (Microsoft, Seattle, WA). P values less than 0.05 were considered significant.

The time-course profiles for the cell count data from the nonhuman primate studies were estimated within the framework of two-way multiplicative linear mixed effects analysis of co-variance model [56]. The co-variables in the model were treatment (saline vs. rhGH) and time. The latter was added based on a natural spline basis [*57*] with knots at times 0, 16, 23, 31, and 60 days. A random effect term was used to account for inter-subject variability. An auto-correlation dependence structure of order 1 was assumed among the measurement errors for each subject. These analyses were carried out using the R [*58*] extension packages nlme and spline. An outlier in the rhGH-treated group was excluded from the statistical analyses.

## Supporting Information

Figure S1 rhGH-mediated mitigation against lethal irradiation is not restricted to BALB/c mice. C57BL/6 mice were irradiated with 9 Gy and treated with rhGH i.v. daily at a dose of 20 µg/dose/day for 5 days. rhGH was given within one hour post irradiation. The bars represent the period of the time that the animals were treated. rhGH stands for recombinant human growth hormone.
(0.08 MB TIF)

Table S1 Effect of rhGH on blood citruline level. BALB/c mice were irradiated with 7.5 Gy and treated with rhGH a for 5 days. Plasma was harvested at day 3 and day 14 post irradiation. The concentration of citruline was determined by high-performance liquid chromatography. The baseline level of citruline in normal BALB/c mice was 61.8±5.7 µg/ml plasma. Each group contains 7 animals. This is a representative of two experiments. rhGH stands for recombinant human growth hormone.
(0.04 MB DOC)
