# Supplementary figures and images for: Growth Hormone Mitigates against Lethal Irradiation and Enhances Hematologic and Immune Recovery in Mice and Nonhuman Primates
Source: PLoS One. 2010 Jun 16;5(6):e11056. doi: 10.1371/journal.pone.0011056 (PMC2886847; doi:10.1371/journal.pone.0011056)

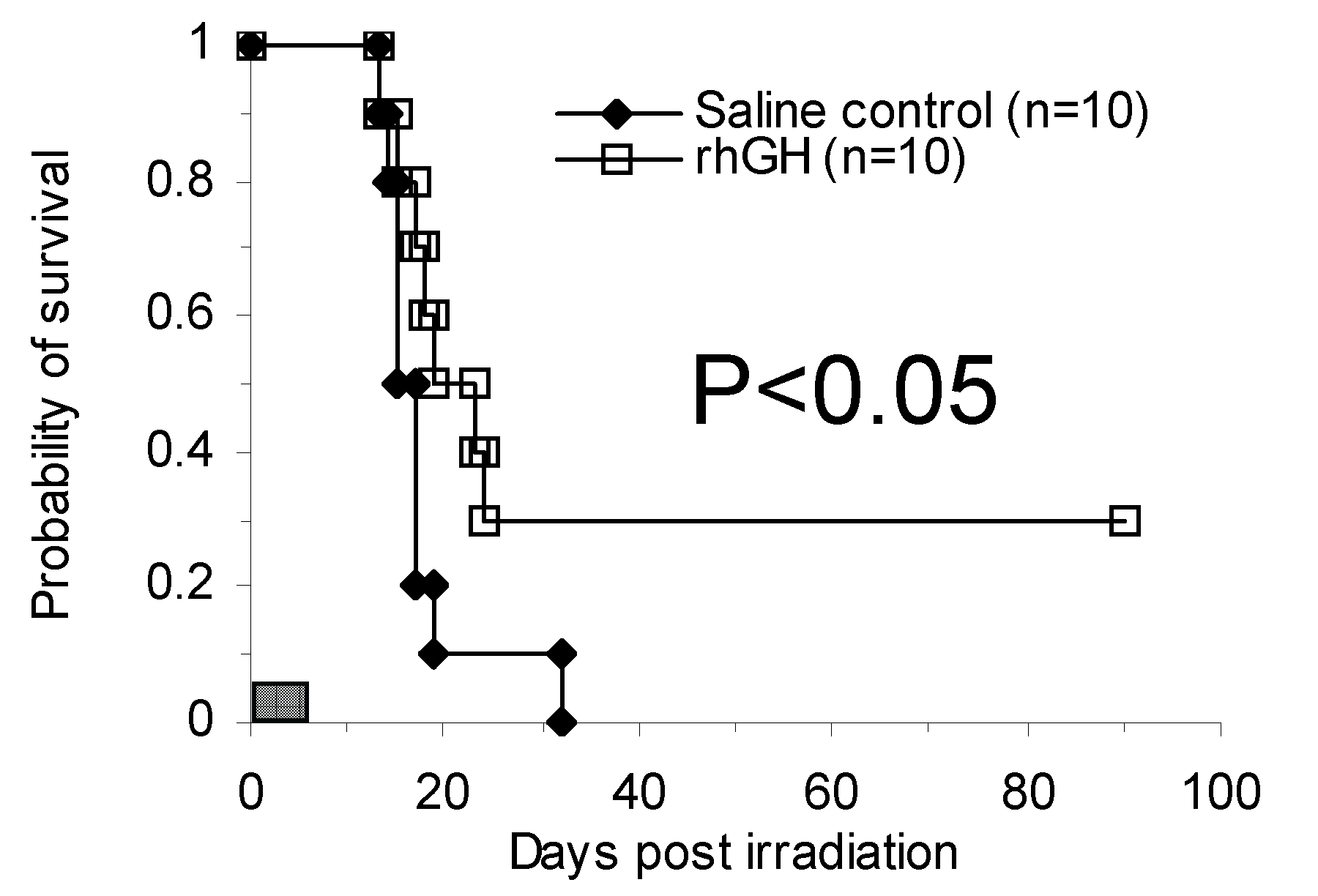

Supplement: Figure S1 — rhGH-mediated mitigation against lethal irradiation is not restricted to BALB/c mice. C57BL/6 mice were irradiated with 9 Gy and treated with rhGH i.v. daily at a dose of 20 µg/dose/day for 5 days. rhGH was given within one hour post irradiation. The bars represent the period of the time that the animals were treated. rhGH stands for recombinant human growth hormone. (0.08 MB TIF) [file pone.0011056.s001.tif]
