# Supplementary material for: Growth Hormone Mitigates against Lethal Irradiation and Enhances Hematologic and Immune Recovery in Mice and Nonhuman Primates
Source: PLoS One. 2010 Jun 16;5(6):e11056. doi: 10.1371/journal.pone.0011056 (PMC2886847; doi:10.1371/journal.pone.0011056)
Supplement: Table S1 — Effect of rhGH on blood citruline level. BALB/c mice were irradiated with 7.5 Gy and treated with rhGH a for 5 days. Plasma was harvested at day 3 and day 14 post irradiation. The concentration of citruline was determined by high-performance liquid chromatography. The baseline level of citruline in normal BALB/c mice was 61.8±5.7 µg/ml plasma. Each group contains 7 animals. This is a representative of two experiments. rhGH stands for recombinant human growth hormone. (0.04 MB DOC) [file pone.0011056.s002.doc]

Table S1. Effect of rhGH on blood citruline level

| Groups | Citruline (μg/ml plasma) | |
| --- | --- | --- |
|  | Day +3 | Day +14 |
| Saline control | 17.8±10.3 | 53.2±16.6 |
| rhGH | 21.2±3.0 | 55.0±11.9 |

BALB/c mice were irradiated with 7.5 Gy and treated with rhGH a for 5 days. Plasma was harvested at day 3 and day 14 post irradiation. The concentration of citruline was determined by high-performance liquid chromatography. The baseline level of citruline in normal BALB/c mice was 61.8±5.7 μg/ml plasma. Each group contains 7 animals. This is a representative of two experiments. rhGH stands for recombinant human growth hormone.
